# Supplementary material for: MRI Radiomics and Predictive Models in Assessing Ischemic Stroke Outcome—A Systematic Review
Source: Diagnostics (Basel). 2023 Feb 23;13(5):857. doi: 10.3390/diagnostics13050857 (PMC10000411; doi:10.3390/diagnostics13050857)
Supplement: Supplementary file 1 [file diagnostics-13-00857-s001.zip › diagnostics-2221631-supplementary.pdf]

Supplementary Materials

Table S1. PRISMA checklist

Section/topic	#	Checklist item	Reported on page #
**TITLE**			
Title	1	Identify the report as a systematic review, meta-analysis, or both.	p. 1 (manuscript)
**ABSTRACT**			
Structured summary	2	Provide a structured summary including, as applicable: background; objectives; data sources; study eligibility criteria, participants, and interventions; study appraisal and synthesis methods; results; limitations; conclusions and implications of key findings; systematic review registration number.	p. 1 (manuscript)
**INTRODUCTION**			
Rationale	3	Describe the rationale for the review in the context of what is already known.	p. 2-3 (manuscript)
Objectives	4	Provide an explicit statement of questions being addressed with reference to participants, interventions, comparisons, outcomes, and study design (PICOS).	p. 3 (manuscript)
**METHODS**			
Protocol and registration	5	Indicate if a review protocol exists, if and where it can be accessed (e.g., Web address), and, if available, provide registration information including registration number.	p. 3 (manuscript)
Eligibility criteria	6	Specify study characteristics (e.g., PICOS, length of follow-up) and report characteristics (e.g., years considered, language, publication status) used as criteria for eligibility, giving rationale.	p. 3 (manuscript)
Information sources	7	Describe all information sources (e.g., databases with dates of coverage, contact with study authors to identify additional studies) in the search and date last searched.	p. 3 (manuscript)
Search	8	Present full electronic search strategy for at least one database, including any limits used, such that it could be repeated.	p. 3 (Supplementary materials - Table S2)
Study selection	9	State the process for selecting studies (i.e., screening, eligibility, included in systematic review, and, if applicable, included in the meta-analysis).	p. 3-4 (manuscript)
Data collection process	10	Describe method of data extraction from reports (e.g., piloted forms, independently, in duplicate) and any processes for obtaining and confirming data from investigators.	p. 3-4 (manuscript)

Data items	11	List and define all variables for which data were sought (e.g., PICOS, funding sources) and any assumptions and simplifications made.	p. 3-4 (manuscript)
Risk of bias in individual studies	12	Describe methods used for assessing risk of bias of individual studies (including specification of whether this was done at the study or outcome level), and how this information is to be used in any data synthesis.	p. 4 (manuscript)
Summary measures	13	State the principal summary measures (e.g., risk ratio, difference in means).	p. 4 (manuscript)
Synthesis of results	14	Describe the methods of handling data and combining results of studies, if done, including measures of consistency (e.g., I^2) for each meta-analysis.	p. 4 (manuscript)

Section/topic	#	Checklist item	Reported on page #
Risk of bias across studies	15	Specify any assessment of risk of bias that may affect the cumulative evidence (e.g., publication bias, selective reporting within studies).	p. 4 (manuscript)
Additional analyses	16	Describe methods of additional analyses (e.g., sensitivity or subgroup analyses, meta-regression), if done, indicating which were pre-specified.	p. 4 (manuscript)
**RESULTS**			
Study selection	17	Give numbers of studies screened, assessed for eligibility, and included in the review, with reasons for exclusions at each stage, ideally with a flow diagram.	p. 4-5 (manuscript)
Study characteristics	18	For each study, present characteristics for which data were extracted (e.g., study size, PICOS, follow-up period) and provide the citations.	p. 4-7 (manuscript) & p. 4 (supplementary materials)
Risk of bias within studies	19	Present data on risk of bias of each study and, if available, any outcome level assessment (see item 12).	p. 5 (manuscript) & p. 4 (Supplementary materials – Table S3)
Results of individual studies	20	For all outcomes considered (benefits or harms), present, for each study: (a) simple summary data for each intervention group (b) effect estimates and confidence intervals, ideally with a forest plot.	p. 4-8, table 2 and figures 2 and 3 (manuscript)
Synthesis of results	21	Present results of each meta-analysis done, including confidence intervals and measures of consistency.	-
Risk of bias across studies	22	Present results of any assessment of risk of bias across studies (see Item 15).	p. 7-8 and figure 3 (manuscript)
Additional analysis	23	Give results of additional analyses, if done (e.g., sensitivity or subgroup analyses, meta-regression [see Item 16]).	p. 7-8 and figure 2 (manuscript)
**DISCUSSION**			
Summary of evidence	24	Summarize the main findings including the strength of evidence for each main outcome; consider their relevance to key groups (e.g., healthcare providers, users, and policy makers).	p. 9-11 (manuscript)
Limitations	25	Discuss limitations at study and outcome level (e.g., risk of bias), and at review-level (e.g., incomplete retrieval of identified research, reporting bias).	p. 11 (manuscript)
Conclusions	26	Provide a general interpretation of the results in the context of other evidence, and implications for future research.	p. 11 (manuscript)

**FUNDING**			
Funding	27	Describe sources of funding for the systematic review and other support (e.g., supply of data); role of funders for the systematic review.	-

From: Moher D, Liberati A, Tetzlaff J, Altman DG, The PRISMA Group (2009). Preferred Reporting Items for Systematic Reviews and Meta-Analyses: The PRISMA Statement. PLoS Med 6

Table S2. Search strategies

PubMed	Embase
("stroke"[MeSH Terms] OR "stroke"[All Fields] OR "strokes"[All Fields] OR "stroke s"[All Fields]) AND "radiomic*"[All Fields] AND ("magnetic resonance"[All Fields] OR "MRI"[All Fields])	('stroke'/exp OR 'stroke') AND ('radiomics'/exp OR 'texture analysis'/exp OR texture) AND (‘magnetic resonance’/exp OR MRI)

Table S3. Radiomics workflow main steps of included studies

Study, year,	Sample size	Data source	Sequences used for FE	Segmentation	Features number and types	Feature selection (FE)	Classification method	Validation method	Model applied to a separate dataset
Quan et al [33], 2021, R	110	Multi center	FLAIR, ADC	manually, 3D, ITK-SNAP	753, first- and second- order statistics	Wilcoxon test, Spearman's correlation, LASSO	MLR	cross-validation	Yes, in 2 different centers training set=110 patients validation set=80 patients
Wang et al [34], 2021, R	598	Single center	DWI	manually, ITK-SNAP	402, first- and second- order statistics	mRMR, LASSO	MLR	10-fold cross-validation	Yes, in the same center training set=399 patients validation set=199 patients
Zhang et al [36], 2022, R	103	Single center	DWI, ADC	manually, ITK-SNAP	234, first- and second- order statistics, wavelet, LG transform	ICCs, Man Whitney U test, mRMR	MLR	10-fold cross-validation	No
Zhou et al [35], 2022, R	522	Single center	DWI, ADC	manually, ITK-SNAP	1310, first- and second- order statistics, wavelet, LG transform,	Spearman's correlation, mRMR, LASSO, SO-LR	MLR	10-fold cross-validation	Yes, the same center training set=311 patients validation set=211 patients
Wang et al [37], 2022, R	1003	Single center	DWI, ADC	automatically, 2D U-Net	513, first- and second- order statistics, wavelet	Sparse representation	RNN	no	Yes, in the same center training set=157 patients validation set=846 patients
Wang et al [38], 2020, R	116	Single center	FLAIR, ADC	manually	15, second-order statistics	NR	MLR	NR	No

R=retrospective study, LASSO=Least absolute shrinkage and selection operator, ITK-SNAP=Insight Segmentation and Registration Toolkit-ANAP, 3D= 3D slicer software, MLR=multivariate logistic regression analysis, Mrmr=minimum redundancy maximum relevance, LG transform= Laplacian of Gaussian transformation, ICCs=Intra- and inter-class correlation coefficients, SO-LR=selection operator logistic regression, RNN=recurrent neural network

Table S4. RQS domains and items

Domain and items	Points
**Domain 1**	**0 to 5 p**
Protocol quality: well-documented image protocols (for example, contrast, slice thickness, energy, etc.) and/or usage of public image protocols allow reproducibility/replicability	2
Multiple segmentations: segmentation by different physicians/algorithms/software, perturbing segmentations by (random) noise, segmentation at different breathing cycles. Analyze feature robustness to segmentation variabilities	1
Phantom study on all scanners - detect inter-scanner differences and vendor-dependent features. Analyze feature robustness to these sources of variability	1
Imaging at multiple time points: collect images of individuals at additional time points. Analyze feature robustness to temporal variabilities (for example, organ movement, organ expansion/shrinkage)	1
**Domain 2**	**-8 to 8 p**
Feature reduction or adjustment of multiple testing decreases the risk of overfitting. Overfitting is inevitable if the number of features exceeds the number of samples. Consider feature robustness when selecting features	-3 or 3
Validation: the validation is performed without retraining and without adaptation of the cut-off value, provides crucial information with regard to credible clinical performance	-5, 2, 3, 4, or 5
**Domain 3**	**0 to 6 p**
Multivariate analysis with non-radiomics features (for example, EGFR mutation) - is expected to provide a more holistic model. Permits correlating/inferencing between radiomics and non-radiomics features	1
Biological correlates demonstration of phenotypic differences (possibly associated with underlying gene–protein expression patterns) deepens understanding of radiomics and biology	1
Comparison to gold standard assess the extent to which the model agrees with/is superior to the current 'gold standard' method (for example, TNM-staging for survival prediction). This comparison shows the added value of radiomics	2
Potential clinical utility report on the current and potential application of the model in a clinical setting (for example, decision curve analysis).	2
**Domain 4**	**0 to 5 p**
Discrimination statistics report discrimination statistics (for example, C-statistic, ROC curve, AUC) and their statistical significance (for example, p-values, confidence intervals). One can also apply resampling method (for example, bootstrapping, cross-validation)	2
Calibration statistics report calibration statistics (for example, Calibration-in-the-large/slope, calibration plots) and their statistical significance (for example, P-values, confidence intervals). One can also apply resampling method (for example, bootstrapping, cross-validation)	2
Cut-off analysis determine risk groups by either the median, a previously published cut-off or report a continuous risk variable. Reduces the risk of reporting overly optimistic results	1
**Domain 5**	**0 to 8 p**
Prospective study registered in a trial database - provides the highest level of evidence supporting the clinical validity and usefulness of the radiomics biomarker	7
Cost-effective analysis report on the cost-effectiveness of the clinical application (for example, QALYs generated)	1
**Domain 6: open science and data** make code and data publicly available. Open science facilitates knowledge transfer and reproducibility of the study	**0 to 4 p**

Table S5. RQS scores for all included studies

Study	PQ	m-Seg	Ph study	Multiple time points imaging	FE	m-var analysis (non-RA features)	Biological correlates	Cut-off analysis	D-stat	C-stat	Pros study	V	Comparison with gold standard	Clinical utility	Cost-effectiveness analysis	Open science and data	RQS total
Quan et al [31]	2	1	0	0	3	1	0	0	1	1	0	4	0	2	0	1	16
Zhang et al [33]	2	1	0	0	3	1	0	0	1	1	0	0	0	2	0	0	11
Wang et al [32]	2	1	1	0	3	1	0	0	1	2	0	2	0	2	0	0	15
Zhou et al [34]	2	1	0	0	3	1	0	0	1	2	0	2	0	2	0	1	15
Wang et al [35]	2	1	0	0	3	1	0	0	2	0	0	2	0	2	0	2	15
Wang et al [17]	2	1	0	0	0	0	0	0	1	0	0	0	0	2	0	0	6

PQ=protocol quality, m-Seg=multiple segmentation, Ph study=phantom study, FE=feature reduction, m-var analysis=multivariable analysis, D-stat=discriminative statistics, C-stat=calibration statistics, Pros study=prospective study, V=validation, RA=radiomics analysis, RQS=radiomics quality score

Table S6. PROBAST scores for all included studies

Study	Risk of bias (ROB)				Applicability (A)			Overall	
	Participants	Predictors	Outcome	Analysis	Participants	Predictors	Outcome	ROB	A
Quan et al [31]	-	+	+	+	-	+	+	+	+
Zhang et al [33]	+	+	+	-	+	+	+	+	+
Wang et al [32]	+	+	+	+	+	+	+	+	+
Zhou et al [34]	-	+	+	+	-	+	+	+	+
Wang et al [35]	-	+	+	+	-	+	+	+	+
Wang et al [17]	+	?	-	-	+	-	-	-	-
